# Validation of the Crystallography Open Database using the Crystallographic Information Framework

**DOI:** 10.1107/S1600576720016532

**Published:** 2021-02-14

**Authors:** Antanas Vaitkus, Andrius Merkys, Saulius Gražulis

**Affiliations:** a Department of Protein–DNA Interactions, Institute of Biotechnology, Life Sciences Center, Vilnius University, Saulėtekio al. 7, LT-10257, Vilnius, Lithuania; b Faculty of Mathematics and Informatics, Vilnius University, Naugarduko g. 24, LT-03225, Vilnius, Lithuania

**Keywords:** Crystallography Open Database, Crystallographic Information Framework, CIF validation, CIF dictionary, DDLm

## Abstract

Data curation practices of the Crystallography Open Database (COD) are described with additional focus being placed on the formal validation using the Crystallographic Information Framework (CIF). The *cif_validate* program, capable of validating CIF files against both the DDL1 and the DDLm dictionaries, is presented and used to process the entirety of the COD. Validation results collected from over 450 000 CIF files are demonstrated to be a useful resource in the data maintenance process as well as the development of the underlying ontologies. A set of programs intended to aid in the dictionary migration from DDL1 to DDLm is also presented.

## Abstract 2

Data curation practices of the Crystallography Open Database are described with greater focus being placed on the *cif_validate* program, capable of validating crystallographic information files against both DDL1 and DDLm dictionaries.

## 1. Introduction   

Large-scale analysis of data can often provide insight into phenomena that are not obvious from individual experiments. For this reason, all scientific data should ideally be made open and readily accessible by programmatic means (Wilkinson *et al.*, 2016 ▸). Open databases put this idea into practice by organizing and presenting data sets of various origins in a uniform way (Berman *et al.*, 2003 ▸; Vrandečić & Krötzsch, 2014 ▸). However, merely collecting the data should be viewed as insufficient both for databases and for individual researchers. Sound scientific conclusions must always be preceded by a rigorous validation of the data.

Data validity can be divided into three levels. The first level covers the syntactic correctness. Data files that do not strictly adhere to the specified syntax are very likely to be rejected or misinterpreted by the processing software. The second level deals with semantic validity, that is, the conformance to a set of formal requirements for each data field and its relations to other data fields. The third level involves passing more specialized tests that are usually based on heuristics specific to the field of investigation or even require the expertise of an experienced human operator.

It is common practice to express syntactic and semantic constraints using separate specifications. Probably the best known example of such an approach is the Extensible Markup Language (XML) and the XML Schema Definition Language (XSD) (Gao *et al.*, 2012 ▸; Peterson *et al.*, 2012 ▸). XML defines the syntax of the data files while XSD provides a formal way of describing the data. Concepts such as the overall hierarchy of the document, relations between its elements and data types are covered by XSD. A similar separation of concerns is manifested by the JavaScript Object Notation (JSON) file format and the accompanying JSON schema (JSON, 2019 ▸). In the field of crystallography, however, the Crystallographic Information Framework (CIF) (Hall & McMahon, 2006 ▸; Brown & McMahon, 2002 ▸) is preferred despite the widespread popularity of the aforementioned data formats. Released several years prior to XML 1.0, it implements separation of syntax and semantics in the form of the CIF file format and CIF dictionaries (McMahon, 2012 ▸).

The term crystallographic information file refers to a file format family that consists of CIF 1.1 (Hall *et al.*, 1991 ▸) and CIF 2.0 (Bernstein *et al.*, 2016 ▸). Although these formats are mutually incompatible, they do share a lot of common features: both are human readable, store data in an order-independent fashion and use data items as the basic building blocks. A data item itself consists of a data name and associated data values; in the simplest case it can be viewed as a key–value pair. Alternatively, several data items can be grouped together to form multiple packets of related data values. CIF denotes such packets with the loop_ keyword; therefore these groupings are commonly referred to as ‘looped lists’. The recently released CIF 2.0 introduced several modern features such as complex data structures and Unicode support; these changes were also addressed by the latest generation of CIF dictionaries.

CIF dictionaries employ the Dictionary Definition Language (DDL) to describe the semantics of the data and can be classified into three generations based on the DDL version to which they conform [DDL1 (Hall & Cook, 1995 ▸; Hall, 2006 ▸), DDL2 (Westbrook & Hall, 2006 ▸) or DDLm (Spadaccini & Hall, 2012 ▸)]. Some aspects of the semantics in CIF dictionaries are expressed formally in a computer-readable way; others are semi-formal in the sense that the descriptions themselves are machine parsable but the content is intended to be interpreted by a human. Although this arrangement limits the scope of examination that can be carried out in a domain-independent automated fashion, a generalized validator can still detect many irregularities that would otherwise go unnoticed. As a result, formal validation using CIF should be viewed as an essential step of crystallographic data quality assurance.

One of the common criticisms expressed towards the open data community is that the main focus is put on making the data open rather than ensuring the quality and reusability (Williams & Ekins, 2011 ▸; Longo & Drazen, 2016 ▸; Levy & Johns, 2016 ▸; Chen *et al.*, 2018 ▸). As the maintainers of an open scientific data repository, the Crystallography Open Database (COD) (Gražulis *et al.*, 2009 ▸, 2012 ▸), we feel obliged to address these concerns in a constructive manner; providing a detailed description of software and methods employed in our ongoing task of data curation seems to be a suitable approach. Not only does it tackle the issue in the most direct way but it also presents several open-source tools that might prove useful in data validation tasks outside of the COD. In addition, validation messages collected from over 450 000 COD entries are shown to be a valuable resource for both the programmers of CIF-related software and the maintainers of CIF dictionaries.

## 2. Software and methods   

### 2.1. Syntactic analysis   

Crystallographic information in the COD is stored using the CIF 1.1 file format. To ensure the syntactic correctness of these files our team has developed the *COD::CIF::Parser* error-correcting CIF parser which was shown to be one of the fastest and most complete in the field (Merkys *et al.*, 2016 ▸). The parser is actively maintained and has been recently extended to support the CIF 2.0 format. Bindings for C, Perl and Python programming languages are readily available in the most recent releases of Debian (Merkys, 2021*a* ▸) and Ubuntu (Merkys, 2021*b* ▸) operating systems; Python bindings are also installable using the Python Package Index (PyPI) (Merkys, 2021*c* ▸).

### 2.2. Formal semantic validation   

Formal semantic validation of CIF files against CIF dictionaries is generally carried out using a domain-agnostic computer program known as a validator. The validator used in the COD was developed in-house as a stand-alone *cif_validate* program. It was designed to run as a Unix filter and as such can take its input from both the standard input stream (stdin) and a list of CIF files. Validation results are output to the standard output stream (stdout) and follow the same formal syntax as the warning messages issued by the *COD::CIF::Parser* (Merkys *et al.*, 2016 ▸). This in turn facilitates the automatic parsing and classification of the validation results using external means. Several examples on how the program can be used are provided in the supporting information.

The *cif_validate* program employs the *COD::CIF::Parser* for all of its CIF parsing needs. As a result, it is capable of handling both CIF 1.1 and CIF 2.0 files in combination with the DDL1 and DDLm dictionaries. DDL2 dictionaries are currently not supported; however, this shortcoming is acceptable since the majority of IUCr-curated dictionaries are written in DDL1. The negative impact is lessened even more by the fact that all of the IUCr dictionaries are being actively upgraded to conform to DDLm.

During the validation process it is assumed that all files in the COD were originally created with DDL1 dictionaries in mind. Even though only about 6000 entries contain the AUDIT_CONFORM loop that explicitly specifies their conformity, the assumption can be deemed a reasonable one simply due to the novelty of DDLm and CIF 2.0. However, since DDLm dictionaries are generally ontologically richer than their DDL1 counterparts, employing them in the validation can reveal even more complex data inconsistencies. As a result, entries in the COD are validated against both the DDL1 and the DDLm dictionaries. DDL constraints that the *cif_validate* program is capable of validating are listed in Table 1 ▸.

#### 2.2.1. DDL1 dictionaries   

DDL1 dictionaries arose from the initial effort to describe properties and relations pertaining to chemical crystallography. In 2014 the DDL1 language reached its end of life and was formally deprecated by the IUCr in favour of DDL2 and DDLm (IUCr, 2020*b* ▸). However, due to their widespread adoption in the field of small-molecule crystallography, DDL1 dictionaries were not removed outright and instead entered a gradual phase-out period. The ongoing maintenance of these legacy dictionaries is carried out in a dedicated IUCr GitHub repository (COMCIFS, 2020*c* ▸).

Several of the legacy dictionaries in this repository have already received minor changes. As a result, they are employed in the validation of the COD instead of the ones provided on the IUCr website. Although no additional features were added, it is still worthwhile to provide a more detailed interpretation of the existing DDL1 constraints. This serves both as the documentation of the validator and as a reference to be used in comparison with the DDLm-based validation. The *cif_validate* program validates the following DDL1 constraints:

(i) Data type. Data values must conform to the declared data type. The type choice is limited to an all-encompassing character string or a numeric value.

(ii) Standard uncertainty eligibility. Data values must not be accompanied by standard uncertainty values unless explicitly stated otherwise. This constraint only applies to standard uncertainty values recorded using the concise notation (NIST, 2020 ▸).

(iii) Enumeration set. Data values may be constrained to a predefined value set.

(iv) Permitted range. Data values may be limited to a specific value range. Both numeric and character ranges are supported; however, in practice only the former are encountered.

(v) Looped list eligibility. Data items must be placed in an appropriate looped list context. DDL1 data items are assigned one of three states in regards to their presence in a looped list: yes, no or both. The yes state indicates that the item must only appear in a looped list while the no state indicates that the item must not appear inside a looped list. The both state signifies that the item can appear in any looped list context.

(vi) Looped list keys. Data items that serve as looped list references must all be present in the loop and provide a unique code to each loop packet. As a result, these references are subject to a similar set of constraints to non-null unique composite primary keys in the relational data model.

(vii) Looped list integrity. Data items that share the same looped list reference must appear in the same looped list.

(viii) Looped list category homogeneity. Looped lists must consist only of data items from the same category.

(ix) Referential integrity. Referenced data items must be present and contain all the values of the referencing data items. The relation between a referenced item (parent item) and a referencing item (child item) is most similar to a relation between a candidate key and a foreign key used in the relational database model. The parent–child relation in DDL1 can be specified either in the definition of the parent item, in the definition of the child item or in both. Also note that there is no explicit requirement for the values of the parent data item to be unique.

(x) Deprecation. Deprecated data items should not be used. Data items may be marked as replaced by other data items. The presence of these items in data files is not outright invalid, but highly discouraged. As a result, replacement data items should be used instead of the replaced ones when possible. The simultaneous presence of both the replaced and the replacement data items should also be avoided since it might lead to contradictory data. Data item deprecation check is disabled by default and can be enabled using the --report-deprecated option.

(xi) Mandatory looped list items. Looped lists may be required to contain certain data items.

(xii) Looped list value uniqueness. A set of data items may be required to have a combined unique value.

#### 2.2.2. DDLm dictionaries   

DDLm is the youngest dictionary definition language and is still undergoing active development. It has embraced and improved on features such as strong data typing, complex nested data structures, relational modelling capabilities and the support of embedded methods written in Relational Expression Language for Dictionary Methods (dREL) (Spadaccini *et al.*, 2012 ▸). In light of these enhancements the IUCr has started an ongoing effort to migrate all of the official CIF dictionaries to the DDLm language. However, even though the migration was complemented by the release of the DDLm-compatible CIF 2.0 data format (Bernstein *et al.*, 2016 ▸), the application scope of the redesigned dictionaries is in no way limited to the CIF format and can potentially be used by software requiring different data exchange formats such as XML, JSON or even relational database schemas.

The flavour of DDLm employed by the IUCr differs slightly from that described in the original publication (Spadaccini & Hall, 2012 ▸) because of the changes that were carried out to adapt it to the field of crystallography. Since the primary purpose of *cif_validate* is to validate the crystallographic information files the program was developed in regard to the latest stable release of the DDLm reference dictionary (Bollinger *et al.*, 2020 ▸) available in the official IUCr GitHub repository (COMCIFS, 2020*b* ▸).

The *cif_validate* program currently validates the following DDLm dictionary constraints:

(i) Data type. Data values must conform to the declared data type. DDLm supports nearly 20 data types including several numeric types (*i.e.* Integer, Real), complex string types with an underlying internal syntax (*i.e.* Uri, DateTime, Version) and types specific to the field of crystallography (*i.e.* Symop). Since the data types are not described using formal grammars, the validation rules were derived from the human-readable descriptions provided in the DDLm reference dictionary. In cases where this approach was not sufficient the CIF 2.0 grammar and the work-in-progress dREL specification (Hall *et al.*, 2008 ▸) were consulted.

(ii) Container type. Data values must be stored using an appropriate data structure. DDLm allows specification of whether a data item can be stored using a single value or if a more complex data structure (*i.e.* List, Matrix, Table) needs to be employed.

(iii) Standard uncertainty presence. Measurand data values must be accompanied by the standard uncertainty (s.u.) values. This can be achieved by using either the concise parenthesis notation or a separate data item.

Three types of violations are reported by the validator in regards to this constraint: (*a*) missing s.u. values; (*b*) prohibited s.u. values: presence of s.u. values related to non-measurand data values; (*c*) mismatching s.u. values: s.u. values are provided using both the parenthesis and the separate data item notation, but the corresponding values differ.

The presence of s.u. values is a recent introduction into the set of constraints mandated by the DDL dictionaries and thus renders multiple DDL1-compliant files invalid. To avoid this the --ignore-missing-su option was introduced, which enables the exclusion of this constraint from the validation.

(iv) Enumeration set. Data values may be constrained to a predefined value set. The DDLm enumeration set constraint is analogous to the DDL1 one save for the effect that the declared data type has on the logic required to determine if a value belongs to the given set. For example, the same DateTime value can be represented by multiple text strings with the differences ranging from as trivial as the letter capitalization to as complex as the time zone offset.

(v) Permitted range. Data values may be limited to a specific value range. The DDLm value range constraint is most similar to the DDL1 one, although slightly less formally defined in regards to the allowed data types and the range syntax itself. As a result, it was decided to implement the range constraint based on its current usage in the IUCr dictionaries, that is, as pertinent to numbers with the lower bound and the upper bound being mutually optional.

(vi) Looped list eligibility. Data items must be placed in an appropriate looped list context. In DDLm, eligibility to appear in a looped list is not defined as a property of an individual data item but rather depends on a property of the parent category called a class (_def inition.class). The Loop class indicates that data items from the category may appear in a looped list while the Set class indicates that data items from the category must not appear in a looped list.

(vii) Category integrity. Data items from the same looped category must all reside in the same looped list. However, there is an exception to this rule. DDLm allows definition of tables with sparsely populated columns as two separate looped categories, with one category acting as the parent of the other (*i.e.* the ATOM_SITE and ATOM_SITE_ANISO categories in the CIF_CORE dictionary). In cases like these data items from the child category are allowed to reside in the data loop of the parent category.

(viii) Category key properties. Data items that comprise the category key must all be present in the looped list and provide a unique code to each loop packet. The following types of key constraint violations are reported: (*a*) incomplete key: one or more data items comprising the key are not given and neither a default value nor a dREL method is provided to generate a substitute value; (*b*) duplicate key values: at least two normalized key values are identical.

Since the validator currently does not execute dREL methods, any missing key data item with a value evaluation method is silently ignored.

(ix) Referential integrity. Referenced data items must be present and contain all the values of the referencing data items. DDLm data item references are subject to the same constraints as the DDL1 ones while also being implemented in a simpler manner. DDLm allows specification of these relations only in the definition of the referencing data item as opposed to the multitude of options provided by the parent–child relation of the DDL1.

(x) Deprecation. Deprecated data items should not be used. DDLm allows the deprecation of data items by marking them as being replaced by other data items. This replacement mechanism is designed to be used in a more formal way than the DDL1 one. That is, DDL1 employs a similar mechanism both for renaming and for deprecating data items whereas the DDLm mechanism is strictly reserved for definitions that have been deemed deficient in some way. Data item deprecation check is disabled by default and can be enabled using the --report-deprecated option.

(xi) Data item aliasing. Data values associated with synonymous data names must match. DDLm provides a mechanism to easily assign several data names to a single data item definition with no one name having precedence over the others. This approach to data item aliasing proves extremely useful in dictionary management tasks; however, it also inadvertently introduces a risk of data anomalies. For instance, a data file can be easily rendered ambiguous by simply introducing an aliased data item with a value that differs from the value of its counterpart. On the other hand, the simultaneous presence of aliased data items can sometimes result from a deliberate decision, for example, as a measure to retain compatibility with legacy software. With this in mind, aliased data items are only reported if the normalized data values do not match. Validation of looped aliased values is currently not supported.

(xii) Application scope. Dictionary data items must appear in the appropriate application scope. DDLm requires all dictionary files to adhere to the data item application scope constraints specified in the DDLm reference dictionary. The constraint establishes which data items are mandatory, recommended or prohibited in the specified dictionary context (Dictionary, Category, Item). As a result, inspection of the application scope is only carried out when validating a dictionary file against the DDLm reference dictionary.

#### 2.2.3. Validation of concatenated enumeration sets   

Evolution is a natural step in the ontology life cycle (Ashraf *et al.*, 2015 ▸) and the CIF_CORE dictionary is no exception. With the maintenance period of 27 years and more than 100 revisions, various approaches to data management have been explored and later on retired. However, in the world of ontologies ‘deprecated’ does not mean ‘unused’, and certain features need to be supported long after they have been deemed inadequate.

**Figure 1:**
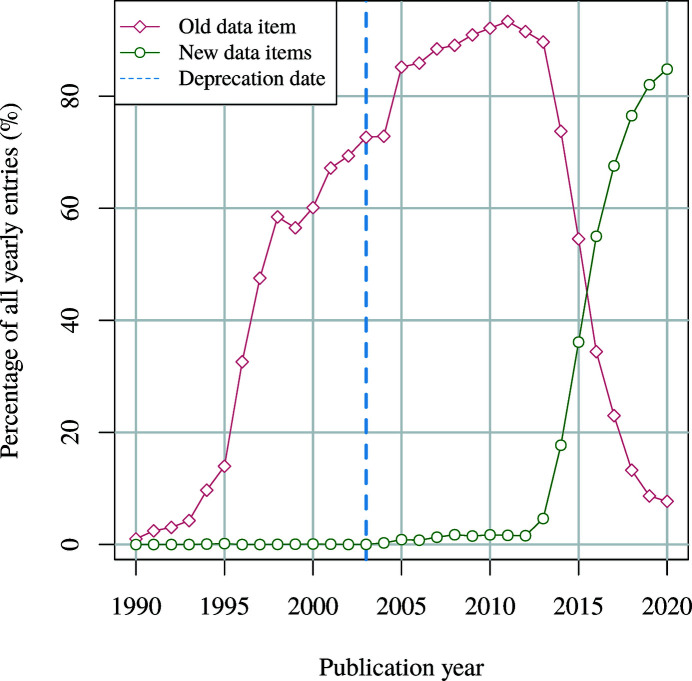
The prevalence of the _atom_site_refinement_flags and its replacement data items in the COD (revision 249495) grouped by publication year.

Concatenated enumeration sets is one such legacy feature. Normally, a DDL enumeration set is treated as a list of all permissible values for a given data item; however, in the case of the _atom_site_ref inement_flags data item this notion was expanded upon by allowing value concatenation. It was declared in the human-readable part of the definition that the valid values are not limited to the listed ones (S, G, R, D, T, U, P, .) but also include various combinations of these values (*i.e.* PR, PDU, DUP). Because of this deviation from the standard dictionary practices as of CIF_CORE version 2.3 (28 September 2003), the _atom_site_ref inement_flags data item is considered deprecated and was replaced by a set of well structured data items (IUCr, 2003 ▸). Despite that, an inspection of more than 450 000 CIF files from the COD has revealed that the data item is still widely in use (Fig. 1 ▸).

In order to correctly handle concatenated enumeration values a special mode was implemented in the validator. By default, this mode is enabled for the _atom_site_refinement_flags data item; however, the list of the affected data items can be modified using the --treat-as-set option.

#### 2.2.4. dREL-based validation   

DDLm dictionaries can optionally contain snippets of code written in dREL. These dREL scripts enhance the dictionaries by providing algorithmic means of calculating or validating data values based on other data items. In addition, the scripts can be employed to conditionally modify the data definitions by assigning attributes based on the contents of the validated file.

To make use of all of the dREL features a dedicated interpreter has to be built first. This task is greatly simplified by the existence of an annotated dREL grammar (COMCIFS, 2020*a* ▸) and several working implementations such as *JsCifBrowser* and *PyCIFRW* (Hester, 2006 ▸). Despite that, the current version of the *cif_validate* program does not yet support dREL methods. This drawback is somewhat mitigated by the fact that most of the dREL validation methods provided in the CIF_CORE dictionary have already been implemented in the COD validation workflow as domain-specific tests.

### 2.3. Additional domain-specific tests   

Formal semantic validation does not cover the entire variety of domain-specific constraints and as such *ad* *hoc* tests have to be developed. These tests encode the ‘common sense’ a researcher would use to assert that a CIF file is correct. A notable example of such a validation tool in the field of small-molecule crystallography is *PLATON* (Spek, 2003 ▸), which is capable of evaluating molecular geometry and chemistry implied by a crystal structure description. In the COD the *cif_cod_check* program from the *cod-tools* package is used to perform crystallographic data checks based on the IUCr data validation guidelines (IUCr, 2020*a* ▸).

### 2.4. Data validation procedures   

Validation programs are routinely used in the COD data management tasks both as stand-alone tools and as components of larger automated sys­tems. From the moment a CIF file is presented for deposition any changes to the file contents are followed by a set of automated quality tests. The test sets differ depending on the context; however, they invariably include the syntactic analysis.

The entire COD data set is stored in a centralized repository using the *Subversion* version control system. Any changes to the data set are followed by a ‘commit’ request to the central repository where the changes are either accepted or refused. *Subversion* repositories can be set up to execute a specific program called a pre-commit hook prior to each commit. The current version of the COD pre-commit hook runs the *COD::CIF::Parser* on all newly added or modified CIF files and rejects the commit attempt if any parsing errors are detected. As a result, all CIF files in the COD are guaranteed to be syntactically correct.

A certain degree of semantic validity and adherence to domain-specific requirements is ensured by the CIF deposition pipeline accessible via the COD website. The pipeline contains tools that are capable of diagnosing various issues and even correcting the simple ones. In this case, validation issues encountered during the deposition do not terminate the process outright but are rather reported to the user for further inspection. All validation issues are presented in a common error message format and are assigned one of the following severity levels: NOTE, WARNING or ERROR. Notes contain information about suspicious data values or automatically applied data corrections and can generally be ignored if so desired. Warnings and errors, however, signal more serious issues that need to be resolved in order to continue with the deposition.

One may be tempted to include the programs from the pipeline as *Subversion* pre-commit hooks alongside the *COD::CIF::Parser* to ensure an even greater level of data validity. However, while the syntactic correctness can be viewed as the mandatory minimum quality requirement, semantic inconsistencies or even domain-specific constraint violations have to be tolerated to some extent. For example, it is official COD policy to accept all syntactically correct CIF files originating from peer-reviewed journals even if the data do not fully adhere to the COD quality criteria; it would be unreasonable to reject such data since they have already been deemed significant in the eyes of the scientific community. CIF files provided as pre-publication material or as personal communications, however, are subjected to the strictest available COD quality tests.

The same validation tools are also routinely used on the entirety of the COD as part of the quality assurance strategy. Collected validation messages are recorded in a SQL database and manually analysed in order to identify the most common issues as well as to develop software solutions that address them. Analysis of one such routine checkup is presented in Section 3.1.

## 3. Results   

### 3.1. Comparison of DDL1-based and DDLm-based validation   

Formal validation against DDL dictionaries was carried out on revision 249495 of the COD using the *cif_validate* program from the *cod-tools* package version 3.0.0. The entire data set was validated using two different parameter sets which were tailored either to DDL1-based or to DDLm-based ontologies. The DDL1-based validation was run using the default options, while the DDLm-based validation included an additional --ignore-missing-su option. In both cases the dictionary set was limited to the latest available revisions of the CIF_CORE^1^^,^ 2 and CIF_COD^3^ dictionaries. Validation results are summarized in Table 2 ▸.

DDL1-based and DDLm-based validation results differ for several reasons. Some of the differences arise as a consequence of the semantically richer DDLm descriptions, while others stem from the ontological changes implemented during the migration from DDL1 to DDLm. The following main reasons were identified:

(i) Constraint modifications. Several data item definitions have been changed in regards to the enumeration range, enumeration values, s.u. eligibility and loop eligibility. The resulting incompatibilities between the properties of corresponding data items explain the majority of differences in the single-value-based validation group.

(ii) Data type change. The DDLm version of the dictionary uses a much richer type system. As a result, some value constraints that were previously only provided as human-readable descriptions became properly formalized. This change allows the detection of deviations from specified non-numeric data type syntax (*i.e.* Date, DateTime, Symop) as well as differentiation between integer and floating-point numbers in situations where this distinction is desired. Examples of observed illegitimate floating-point value usage include the Miller indices (_exptl_crystal_face_index_*), the oxidation number (_atom_type_oxidation_number) and the number of reflections (_cell_measurement_ref lns_used).

(iii) Case sensitivity. DDL1 character strings are strictly case sensitive while the same is true only for some DDLm data types. On the one hand, this change results in a few instances of value uniqueness loss. On the other hand, it inadvertently repairs several previously broken foreign key relationships.

(iv) Artificial primary keys. Several natural category keys in the DDLm dictionary have been replaced by artificially constructed ones. As a result, the uniqueness constraint was dropped for the data items involved in the natural category key. For example, in the DDL1 version of the dictionary the symmetry operation string (_symmetry_equiv_pos_as_xyz) serves as the key of the SYMMETRY_EQUIV category while in the DDLm version an arbitrary integer value is used as the key instead (_symmetry_equiv_pos_site_id). As a result, duplicate symmetry operators are only reported when validating with the DDL1 dictionary.

(v) Changed category keys. Several composite keys in the DDLm dictionary have been changed to contain additional data items in order to allow the construction of truly unique identifiers. For example, the DDL1 dictionary defines the GEOM_BOND category key as consisting of the atom labels (_geom_bond_atom_site_label_*) whereas the DDLm dictionary extends it by including the atom symmetry site data items (_geom_bond_site_symmetry_*). This type of modification both decreases the number of key uniqueness violations and increases the number of missing key item violations.

(vi) Treatment of partial category keys. Both DDL1 and DDLm require that key data items have a combined unique value, but only DDL1 explicitly states that all items of a composite key must be collectively present. Because of this, the DDL1-based validation only checks the uniqueness of a composite key if all key items are present. Conversely, DDLm-based validation always carries out a composite uniqueness check on the existing key items even if they do not comprise a complete key.

(vii) Additional data item definitions. The DDLm version of the CIF_CORE dictionary has absorbed the definitions of the CIF_SYM symmetry dictionary. Since the symmetry dictionary was written in DDL2, it could not be used in the DDL1-based validation. In addition, the DDLm-based dictionary contains multiple data name aliases that are not present in the DDL1-based dictionary in any form. The combination of these two factors results in a slight decrease in unrecognized data names.

(viii) Complex data structures. Several data item definitions in the DDLm-compliant dictionaries have been assigned data structures that are not supported by DDL1. For example, the _atom_type_scat_versus_stol_list data item that was originally designed to store scattering-factor information in a free-form text field was redefined as a structured list. Even though such changes allow the description of data in a more formal way, they do have the downside of being incompatible with both the DDL1 ontology and the CIF 1.1 data format.

The outlined differences highlight a set of changes that need to be addressed while migrating from DDL1 to DDLm. The overall trend suggests that due to the formalization of certain constraints the DDLm-based validation is capable of detecting additional inconsistencies in the data.

### 3.2. Advice on identifying and correcting issues in CIF files   

Validation of the entire COD provides a useful insight into a CIF data set that was accumulated and maintained over a period of more than 20 years. However, since a variety of semantic problems in the COD are routinely addressed by the database maintainers, such analysis does not allow one to identify all of the most common semantic problems that were present in the original CIF files prior to their deposition to the COD. This drawback was addressed by analysing more than 30 000 CIF files (collectively containing more than 66 000 data blocks) that were provided as supplementary material for publications from various peer-reviewed sources published over the past five years (2016–2020).

The analysis revealed that ∼2% of files contain syntax errors, ∼95% of files raise DDL1 validation issues and ∼98% of files raise DDLm validation issues when validated using the setup detailed in Section 3.1. The following are some of the most common syntax and semantic issues present in the recently published peer-reviewed CIF files:

(i) The use of characters that do not belong to the permitted character set, which in the case of CIF 1.1 files is limited to a subset of the ASCII character set (Hall *et al.*, 2006*a* ▸). While some special characters (*i.e.* Greek letters, accented characters) can be expressed using special codes (*i.e.* ‘\a’ for ‘α’, ‘\"u’ for ‘ü’) (Hall *et al.*, 2006*b* ▸), the majority of Unicode characters are not directly supported at all. However, such characters still tend to appear in author names, experiment descriptions or other free-text fields of CIF 1.1 files.

(ii) Omission of the mandatory _space_group_symop_id data item. This data item allows one to assign unique identifiers to symmetry operations provided using the _space_group_symop_operation_xyz data item. Although these identifiers almost always take the form of sequential positive integers with the ‘x,y,z’ operation being assigned the ‘1’ identifier, generally one should not automatically extrapolate them from the order of symmetry operations in the SPACE_GROUP category loop. The order of data packets in a CIF looped list does not hold any special meaning and therefore should be modifiable without affecting the interpretation of the file contents. Since the _space_group_symop_id data item is used in the construction of other identifiers (*i.e.* the _geom_bond_site_symmetry_1 data item), explicitly assigning these symmetry operation identifiers guards against the misinterpretation of data upon the possible reorganization of the file.

(iii) Misspelt enumeration values of various data items. The most common mistakes include incorrect capitalization (*i.e.* ‘Monoclinic’ instead of ‘monoclinic’), alternative spellings (*i.e.* ‘whiteish’ instead of ‘whitish’) or incorrect hyphenation (*i.e.* ‘multi scan’ instead of ‘multi-scan’).

(iv) Residual placeholder values. Some publishers provide template files that contain data items with placeholder values intended to ease the construction of supplementary CIF files (*i.e.* the _chemical_absolute_configuration data item with the ‘CHOOSE rm ad rmad syn or unk’ value). However, the use of such templates sometimes also inadvertently leads to the creation of semantically incorrect CIF files in which only some of the placeholder data items have been reassigned proper data values. Care should be taken to double-check and update or remove placeholder data items that are not relevant to the reported structures.

(v) Non-numeric values of the _chemical_melting_point data item. Such values often incorrectly contain explicit measurement units (*i.e.* ‘24 C’, ‘298.15 K’) or provide a value range instead of a single value (*i.e.* ‘136–145’). The value of the _chemical_melting_point data item and most other data items that record temperature measurements must be provided in kelvins and without a unit designator (*i.e.* ‘298.15’). In cases when only a temperature range is available, the _chemical_melting_point_gt and _chemical_melting_point_lt data items should be used to record the lower and the upper range bounds, respectively.

(vi) Non-numeric values of the _exptl_crystal_density_meas data item. Such values often indicate that the density measurement was not carried out at all (*i.e.* ‘Not measured’, ‘None’, ‘n/a’). This type of information can be properly expressed in a uniform way by using CIF special values ‘?’ and ‘.’ which correspond to unknown and inapplicable values, respectively.

(vii) Incorrect usage of CIF special values. According to the CIF syntax, CIF special values are expressed using a single character (either ‘?’ or ‘.’) without any delimiting strings. Despite that, these characters are often incorrectly placed in between single quotes, double quotes or even multiline string delimiters, thus forcing them to be interpreted as text strings consisting of a single question mark or full stop character.

While manually correcting problems in CIF files is feasible and even required in more complex cases, most issues, including the majority of the aforementioned ones, are quite simple and can therefore be addressed using automated means. On the basis of this observation, our team has developed the following tools:

(i) *utf8-to-cif*. Converts UTF-8 text to a form that is compatible with the CIF 1.1 data format. Unicode characters that fall outside the CIF 1.1 character set are preferably expressed as CIF 1.1 special codes with hexadecimal numeric character references being used as a fallback mechanism. Since the use of numeric character references is not a universally accepted approach when dealing with CIF 1.1 files, CIF handling programs that were not developed by the COD team are unlikely to place any special meaning on these references. The program can be used as the initial step in the CIF processing pipeline to avoid syntax errors that may be caused by improperly expressed UTF-8 characters.

(ii) *cif_fix_values*. Resolves various simple semantic issues in CIF files. The program can regularize the values of various temperature data items (*i.e.* the _chemical_melting_point), correct values of the _exptl_crystal_density_meas data item, correct misspelt values by consulting a built-in table or an external replacement list file, and carry out various other minor corrections.

(iii) *cif_correct_tags*. Corrects misspelt data names in CIF files. The program can restore the proper data name by applying several *ad* *hoc* rules and by consulting a built-in table or an external replacement list file.

Several examples of how these programs can be used as a response to validation messages issued by the *cif_validate* program are provided in the supporting information.

### 3.3. Dictionary management tools   

The IUCr initiative to convert the official dictionaries from DDL1 to DDLm has incentivized maintainers of other dictionaries to follow suit. Adopting a new ontology comes with its own set of challenges, which can be made even more difficult by the inclusion of additional requirements such as backward compatibility. Faced with the same problem in regards to the COD-related CIF_COD dictionary our team has developed the following tools to ease the DDL-based ontology migration:

(i) *cif_compare_dics*. Checks a pair of DDL1/DDLm-based dictionaries for compatibility-breaking differences. The program reports discrepancies between corresponding enumeration sets, default values, permitted ranges, standard uncertainty eligibility and loop eligibility, as well as data items unique to one of the dictionaries.

(ii) *cif_ddl1_dic_check*. Checks a DDL1-based dictionary against a set of best practices derived from the human-readable portion of the DDL1 reference dictionary, official IUCr publications (Hall & McMahon, 2006 ▸) and public explanatory comments issued by the IUCr (2000 ▸, 2009 ▸). The program reports internal dictionary discrepancies such as missing, incomplete or contradictory definitions, deviations from the naming convention, and incompatibilities with the relational data model.

(iii) *cif_ddlm_dic_check*. Checks a DDLm-based dictionary against a set of best practices derived from the human-readable portion of the DDLm reference dictionary and public explanatory comments issued by the IUCr (2000 ▸, 2009 ▸). The program reports internal dictionary discrepancies such as incomplete definitions, incorrect data item references and incompatible enumeration ranges.

(iv) *cif_ddlm_dic_print*. Pretty-prints a DDLm dictionary file. If requested, the program can resolve the embedded dictionary import statements and combine data from multiple input files into a single DDLm dictionary file.

(v) *ddl1-to-ddlm*. Converts CIF dictionaries from DDL1 to DDLm. The conversion applies conservative heuristics to determine data item types and assign appropriate data item categories. Dictionaries generated by this program can be used as a starting point for transition from DDL1 to DDLm.

(vi) *dic2markdown*. Produces Markdown-formatted descriptions of DDLm dictionaries that can in turn be used to generate HTML or PDF documents intended as user manuals. It is similar in function to the *makedict.pl* program (IUCr, 2020*c* ▸) used to produce *International Tables for Crystallography* Volume G (Hall & McMahon, 2006 ▸) for DDL1 dictionaries. The intermediate Markdown format is also employed to generate online versions of the COD CIF dictionaries (Vaitkus *et al.*, 2021 ▸).

### 3.4. Validation issue database   

Validation issues associated with COD entries are stored in a publicly available SQL database (cod_validation). The database is updated daily by replacing outdated validation issues with issues generated from changed or newly added entries. The collected validation results can be retrieved via a *RestfulDB*-based (Merkys *et al.*, 2021 ▸) RESTful web interface using conventional client programs (*i.e.* various web browsers, cURL) or by directly querying the SQL database. A more detailed description of the database schema as well as instructions on how to query it is provided in the supporting information.

## 4. Discussion   

### 4.1. The importance of formal validation in ensuring FAIR data   

The urgent need to obtain more value from accumulated research data has been recognized by the scientific community and promoted by research funders as well as on the political level (Collins *et al.*, 2018 ▸). The main guiding principles that allow such value enhancement fall under the FAIR (Wilkinson *et al.*, 2016 ▸) acronym – data should be findable, accessible, interoperable and reusable. While the first two principles are more concerned with metadata and technical protocols, interoperability and reusability depend crucially on the quality of the data proper. The initial concerns of interoperability were directed at the standardization of formats and data representation; however, further measures need to be taken to ensure that the contents of the data archives themselves are as reliable as possible at the current state of scientific knowledge. Robust automated syntax checks, validation against formal dictionaries (Adams *et al.*, 2011 ▸) and especially the application of formal ontologies (Hester, 2016 ▸) are among the key actions that have the potential to greatly improve data reusability and interoperability. Reusability can be even further enhanced by employing data analysis pipelines that enable one to automatically reproduce or even improve the collected results as showcased in recent work in the field of macromolecular crystallography (Joosten *et al.*, 2014 ▸; Grabowski *et al.*, 2019 ▸). The discipline of crystallography in general is known for placing great importance on ensuring that data are both reproducible and FAIR, with the development of CIF being recognized as one of the most important achievements towards this goal (Helliwell, 2019 ▸). CIF handling tools developed by our team provide the means to apply validation against DDL dictionaries to individual files as well as existing collections of crystallographic data. Formal discrepancies between data and dictionaries detected during the validation process can then be addressed by either amending the data, extending the dictionaries or noting semantic exceptions, thus both making the data more suitable for large-scale in-depth analysis and driving the development of the underlying ontologies.

### 4.2. Stewardship of small-molecule crystallographic data   

Strict formal validation of more than 450 000 entries from the COD revision 249495 revealed that ∼36% of entries produce no DDL1 validation issues while only ∼4% of entries produce no DDLm validation issues. However, most publicly available CIF files were created with DDL1 in mind and should not be expected to conform to the stricter requirements imposed by DDLm. Although the changed DDLm category keys as well as newly introduced mandatory complex CIF 2.0 data structures are extremely useful in detecting more obscure data anomalies, validation issues related to these changes do not generally prevent the affected files from being used for most applications. When validation issues involving unrecognized data item names, missing category keys and complex data structures are ignored, the number of COD entries without validation issues rises to ∼52% for DDL1 and ∼60% for DDLm. A more detailed breakdown of distinct validation issue distribution per COD entry is provided in the supporting information.

Even though some data inconsistencies might get accidentally introduced during the deposition process, the majority of reported issues can be traced back to the original data. Since the COD data curation policy mandates that changes made to the data must not misconstrue the original intent, resolution of complex issues often requires input from the authors or at least access to the original publication. As both of these approaches require attention from a trained expert and do not guarantee conclusive results, such issues are addressed on a best efforts basis. On the other hand, simple data discrepancies that can be tackled in an automated fashion are routinely identified and corrected. Consequently, a comprehensive log of validation issues becomes a valuable resource since it allows the users to estimate the suitability of each COD entry as well as aiding data maintainers in their data curation tasks.

Previous examination of the validation messages has resulted in the development of computer programs capable of automatically detecting and correcting some of the most common data discrepancies (Gražulis *et al.*, 2012 ▸). Misspelt data names, incorrect enumeration values and improper usage of temperature measurement units are among the addressed issues; however, additional ones are routinely identified as the database grows. For example, the most recent inspection of the logs revealed a tendency to incorrectly record the _dif frn_standards_interval_time data item values together with their measurement units. Because of the simplicity and prevalence of this issue it will also be addressed in an automated fashion. Once the appropriate software modifications are made, the affected files will be reprocessed and the updated software version will be integrated into the COD processing pipeline to prevent the appearance of such discrepancies in the future.

Although great care is taken to ensure the correct behaviour of the software, the negative effects of a coding error cannot be underestimated. The COD mediates this risk by applying changes in a transparent way and meticulously versioning the data. As a rule, a changelog of any automated modifications is both reported to the human operator and recorded in the processed CIF file itself. In the worst-case scenario, the data can always be restored to any of the previous revisions due to the underlying *Subversion* repository.

The use of a version control system is just one example of how the COD was designed with reproducible scientific research in mind. Openness, data provenance and facilitation of reproducible results are some of the core principles that permeate both the data curation policy and the underlying software. As such, although the COD was developed in regards to crystallographic data, the codebase has proven modular enough to be easily adapted to other fields of scientific research.

The most notable example of such code reuse is the Raman Open Database (ROD) (El Mendili *et al.*, 2019 ▸), a resource that aims to apply the COD data curation practices to Raman spectroscopy measurements as well as relate them to crystallographic data. As a newly founded database the ROD was in a unique position to adopt both the CIF 2.0 format and the DDLm-based validation without inconveniencing the existing users. In addition, semantic validity was deemed mandatory and as a result all deposited files are required to conform to the DDLm dictionaries drafted by the ROD Advisory Board. Similar changes will be implemented in the COD once these innovations are embraced by the larger crystallographic community.

Several applications that support the CIF 2.0 file format (Bollinger, 2016 ▸; Hester, 2006 ▸; Hanson, 2010 ▸) as well as those capable of handling DDLm dictionaries (Hester, 2006 ▸) have already been independently developed outside of the COD. Most of them predate the COD implementations and some even provide additional functionality such as DDL2-based validation and advanced handling of dREL methods. Although the COD mainly relies on CIF handling software that was developed in-house and tailored to the specific needs of data curation tasks, the importance of well maintained alternative implementations should not be underestimated. As previously demonstrated with a multitude of CIF 1.1 parsers (Merkys *et al.*, 2016 ▸), an in-depth comparison of different programs can often reveal coding mistakes as well as ambiguities in the underlying specification.

### 4.3. Use of validation messages in ontology maintenance   

Validation results collected from a sufficiently large data set can also serve as a useful resource in the development of related ontologies. While some of the validation issues describe obvious mistakes, others involve data that simply cannot be expressed within the bounds of a given ontology. As such, routine inspection of the validation output is encouraged in order to discover aspects of the underlying ontologies that could be improved upon. Analysis of the COD validation messages identified several possible enhancements to the CIF_CORE dictionary that serve well as examples of the general ontology maintenance approach.

Some of the enhancements are focused on improving the existing data items. As the field of crystallography keeps moving forward it is reasonable to expect changes in the enumeration ranges, the enumeration sets or even the interpretation of certain values. For example, over 8000 instances of the _refine_ls_hydrogen_treatment data item have the ‘riding’ data value that does not belong to the pre­defined enumeration set. As such, this value should be inspected and included in the existing set if deemed significant enough.

Other enhancements require the introduction of new data items. For example, numeric values of certain data items are often incorrectly written together with the ‘less-than’ or ‘greater-than’ sign (*e.g.* ‘<0.001’, ‘>5’) to provide a range of possible values instead of an exact one. The problem can be resolved by introducing complementary *_lt and *_gt data items to denote the lower and upper value bounds accordingly. A similar approach is already employed in the CIF_CORE dictionary for several data items, *e.g.* the _chemical_melting_point data item. Data items that would benefit most from this type of enhancement include _dif frn_standards_decay_%, _refine_ls_shift/su_max and _ref ine_ls_shift/su_mean.

Although all final decisions regarding the ontology lie with its maintainers, involvement of the community can greatly expedite the development process by providing critical feedback. COD validation results serve a similar purpose by providing insight into unofficial ontology extension trends undertaken by the user community that are not visible from individual CIF files.

## 5. Conclusions   

The Crystallography Open Database is an open curated resource that extensively utilizes the Crystallographic Information Framework in the form of CIF files and DDL dictionaries. The entirety of crystallographic data in the COD is ingested, stored and presented using the CIF 1.1 file format. Strict adherence to CIF syntax and automated syntax checking ensure that any changes to the data result in a syntactically correct CIF file. Semantic validity is addressed in a similar fashion by routinely checking CIF files against domain-specific DDL1 dictionaries as well as COD quality criteria. However, differently from the syntax errors, semantic validation issues are not considered fatal and are either automatically corrected or recorded for further analysis. Validation messages collected from over 450 000 COD files are not only instrumental in various data maintenance tasks but also serve as a valuable resource in the development of crystallographic software and the underlying ontologies. In the spirit of open science, the compiled validation issues as well as the software used to identify them are made publicly available.

COD software was recently modified to support several innovations of CIF, namely the CIF 2.0 file format and the DDLm dictionaries. The updated stand-alone tools such as *cif_validate* have been successfully used to process the entirety of the COD. Comparison of validation results based on DDL1 dictionaries with those based on DDLm dictionaries revealed the advantages and potential problems that might arise while transitioning from DDL1 to DDLm. These observations led to the development of additional tools aimed at facilitating the DDL-based ontology migration.

A significant number of issues identified in supplementary crystallographic data of peer-reviewed publications suggests that CIF might be underutilized by the larger scientific community. Wider adoption of CIF validation tools could have a long-lasting positive effect on the quality of scientific data, since problems detected prior to publication have a greater chance of being unambiguously resolved due to the immediate availability of the authors. As a result, researchers, publishers and data curators are encouraged to make greater use of the DDL dictionaries and a variety of open-source CIF tools including the ones provided by the COD.

## 6. Source-code availability   

All of the programs presented in this paper that were developed by the COD team are distributed as part of the *cod-tools* software package under the terms of the LGPL-3 licence. Instructions on how the software package can be retrieved and installed are provided in the supporting information.

## Supplementary Material

Instructions on how to install and run software discussed in the manuscript, instructions on how to query the database that contains additional data relevant to the discussed research topic as well as some additional graphs that were not important enough to be included in the main body of the article. DOI: 10.1107/S1600576720016532/yr5065sup1.pdf

## Figures and Tables

**Table 1:** DDL1 and DDLm constraints that are validated by the *cif_validate* program The dash symbol (–) marks constraints that are not supported by the given DDL version. A more detailed description of each constraint is provided in Sections 2.2.1 and 2.2.2.

	Relevant data items	
DDL constraints	DDL1	DDLm
Data type	_type	_type.contents
Standard uncertainty	_type_conditions	_type.purpose
Enumeration set	_enumeration	_enumeration_set.state
Permitted range	_enumeration_range	_enumeration.range
Looped list eligibility	_list	_def inition.class
Looped list keys/category keys	_list_reference	_category.key_id, _category_key.name
Looped list integrity/category integrity	_list_reference	_name.category_id
Looped list category homogeneity	_category	_name.category_id
Referential integrity	_list_link_parent, _list_link_child	_name.linked_item_id
Deprecation	_related_function	_def inition_replaced.by
Looped list value uniqueness	_list_uniqueness	–
Mandatory looped list items	_list_mandatory	–
Container type	–	_type.container
Data item aliasing	–	_alias.def inition_id

**Table 2:** Comparison of validation issues identified in the COD using DDL1 and DDLm dictionaries Rows in bold contain data about arbitrary groupings of individual issue types, described in the subsequent non-bold rows. The dash symbol (–) marks features that are not applicable to the given DDL version.

	Affected COD entries			
	Number		Percentage (%)	
Validation issue type	DDL1	DDLm	DDL1	DDLm
**Single-value-based validation**	**51846**	**54698**	**11.44**	**12.07**
Value outside the permitted range	17242	20972	3.80	4.63
Value outside the enumeration set	37248	37370	8.22	8.25
Extra standard uncertainty value	814	83	0.18	0.02
**Referential integrity validation**	**61243**	**61309**	**13.51**	**13.53**
Missing foreign key item	46456	46429	10.25	10.25
Missing foreign key value	15220	15312	3.36	3.38
**Category-based validation**	**172242**	**426703**	**38.01**	**94.16**
Non-unique simple key values	3238	3256	0.71	0.72
Non-unique composite key values	109719	7478	24.21	1.65
Missing key item	81592	425629	18.00	93.92
Missing mandatory looped list item	81066	–	17.89	–
Looped list with items from several categories	943	388	0.21	0.09
Compromised category integrity	72	594	0.02	0.13
**Loop eligibility validation**	**37160**	**597**	**8.20**	**0.13**
Unlooped value inside a looped list	745	597	0.16	0.13
Looped value outside of a looped list	36471	–	8.05	–
**Data type constraint validation**	**32211**	**78823**	**7.11**	**17.39**
Non-numeric value	32211	–	7.11	–
Data type constraint violation (Integer)	–	15819	–	3.49
Data type constraint violation (Float)	–	29899	–	6.60
Data type constraint violation (Date)	–	5	–	0.00
Data type constraint violation (DateTime)	–	9406	–	2.08
Data type constraint violation (Symop)	–	510	–	0.11
Forbidden symbol in non-numeric value	–	34711	–	7.66
**Data structure validation**	**–**	**355009**	**–**	**78.34**
Value not placed in a list	–	32	–	0.01
Missing top level container	–	355009	–	78.34
**DDLm-specific validation**	**–**	**1389**	**–**	**0.31**
Mismatching values of aliased data items	–	1389	–	0.31
**Unrecognized data name**	**123972**	**123806**	**27.36**	**27.32**
No validation issues	186507	17605	41.16	3.88

## Footnotes

1 DDL1 version 2.5.3 was retrieved from the official IUCr DDL1 dictionary maintenance repository (COMCIFS, 2020*c* ▸), master branch, commit 13f203a.

2 DDLm version 3.0.13 was retrieved from the official IUCr DDLm dictionary development repository (COMCIFS, 2020*b* ▸), v3.0.13 tag, commit 24f69f0 (Bollinger *et al.*, 2020 ▸).

3 DDL1 version 0.050 and DDLm version 0.3.1 were both retrieved from the svn://www.crystallography.net/cod/cif/dictionaries repository, revision 254590.
