# Supplementary material for: Validation of the Crystallography Open Database using the Crystallographic Information Framework
Source: J Appl Crystallogr. 2021 Feb 14;54(Pt 2):661–72. doi: 10.1107/S1600576720016532 (PMC8056762; doi:10.1107/S1600576720016532)
Supplement: Supplementary file 1 [file j-54-00661-sup1.pdf]

JOURNAL OF
APPLIED
CRYSTALLOGRAPHY

Volume 54 (2021)

Supporting information for article:

Validation of the Crystallography Open Database using CIF

Antanas Vaitkus, Andrius Merkys and Saulius Gražulis

Validation of the Crystallography Open Database using CIF

Supplementary material

Antanas Vaitkus[1*], Andrius Merkys[1] & Saulius Gražulis[1,2]

[1]Department of Protein–DNA Interactions, Institute of Biotechnology, Life Sciences Center, Vilnius University, Saulėtekio al. 7, LT-10257, Vilnius, Lithuania
[2]Faculty of Mathematics and Informatics, Vilnius University, Naugarduko g. 24, LT-03225, Vilnius, Lithuania
[*]*E-mail: antanas.vaitkus90@gmail.com*

November 10, 2020

Contents

1 Installation instructions for the `cod-tools` software package **2**
- 1.1 Subversion repository . . . 2
- 1.2 GitHub repository . . . 2
- 1.3 Debian package . . . 3

2 Usage examples of the `cif_validate` program **3**

3 Usage examples of programs from the `cod-tools` software package **6**
- 3.1 `utf8-to-cif` . . . 6
  - 3.1.1 Usage example 1: conversion of Unicode characters to CIF 1.1 special codes . . . 6
- 3.2 `cif_fix_values` . . . 7
  - 3.2.1 Usage example 1: correction of misspelt enumeration values . . . 7
  - 3.2.2 Usage example 2: correction of temperature values . . . 8
  - 3.2.3 Usage example 3: correction of density values . . . 8
- 3.3 `cif_correct_tags` . . . 9
  - 3.3.1 Usage example 1: correction of misspelt data items using a replacement list file . . . 10

4 The `cod_validation` database **11**
- 4.1 `RestfulDB` based web interface . . . 11
- 4.2 SQL query examples . . . 11

5 Distribution of distinct validation issues per each COD entry **14**

1 Installation instructions for the cod-tools software package

1.1 Subversion repository

The `cod-tools` software package is maintained using a Subversion repository. The package can be installed on a Debian/Ubuntu OS by executing the following `Bash` commands:

1. Check out the v3.0.0 tag release of the `cod-tools` software package:

```
svn checkout svn://www.crystallography.net/cod-tools/tags/v3.0.0 cod-tools
```

2. Go to the check out directory:

```
cd cod-tools
```

3. Install software packages required to run the programs. Dependency installers for some operating systems are provided under `dependencies/` and can be executed as follows:

```
sh dependencies/Ubuntu-20.04/install.sh
```

4. Build and install the `cod-tools` package to the location specified using the `PREFIX` variable. Note, that the specified location must be writable to the user executing the commands:

```
make all
make check
make install PREFIX=/usr/local/install/cod-tools/cod-tools-3.0.0
```

5. Update environment variables to make installed programs and libraries automatically discoverable by the system. Note, that the `COD_TOOLS_PREFIX` variable should be set to the location that was specified during the package installation in step 4 (i.e. `/usr/local/install/cod-tools/cod-tools-3.0.0`):

```
COD_TOOLS_PREFIX=/usr/local/install/cod-tools/cod-tools-3.0.0
export PATH=${COD_TOOLS_PREFIX}/bin:${PATH}
export PERL5LIB=${COD_TOOLS_PREFIX}/lib/perl5/:${PERL5LIB}
export PYTHONPATH=${COD_TOOLS_PREFIX}/usr/local/lib/python${PY3VERSION}/dist-packages:${PYTHONPATH}
```

Where `${PY3VERSION}` is the Python3 version in the X.Y format (i.e. "3.7"). On Debian/Ubuntu systems this version can be found by executing the following command:

```
py3versions --default --version
```

Commands from step 5 can also be copied to the `~/.bashrc` file to automatically execute them each time a new `Bash` shell is started.

1.2 GitHub repository

The `cod-tools` Subversion repository is also mirrored on GitHub. Installation instructions for the Git repository are mostly identical to those provided for the Subversion repository in section 1.1:

1. Clone the `cod-tools` Git repository:

```
git clone https://github.com/cod-developers/cod-tools.git cod-tools
```

2. Go to the working directory:

```
cd cod-tools
```

3. Checkout the v3.0.0 tag release:

```
git checkout tags/v3.0.0
```

4. Repeat steps 3-5 from section 1.1.

1.3 Debian package

The `cod-tools` software package can also be installed from the official Debian 10 and Ubuntu 20.04 repositories:

```
sudo apt-get install cod-tools
```

Note, that since the `cod-tools` packages available in Debian 10 and Ubuntu 20.04 were based on an older `cod-tools` version, they do not contain all of the DDLm tools described in the article. Future releases of the package should contain all of the described programs.

2 Usage examples of the `cif_validate` program

The `cif_validate` program provides an interface of a Unix filter. As such, the input data can be provided as a standard input stream (*stdin*) as well as a list of CIF files. Several examples of running the `cif_validate` program inside a `Bash` shell under the Ubuntu 20.04 GNU/Linux system are provided below. All of the given examples assume the directory tree structure provided in Fig. S1:

- Validate files `1000000.cif`, `1501972.cif` and `2000000.cif` against the `CIF_CORE` DDL1 dictionary:

```
./cod-tools/bin/cif_validate \
    --ddl1-dictionaries ./dictionaries/ddl1/IUCr/cif_core.dic \
    ./data/cod/cif/1000000.cif \
    ./data/cod/cif/1501972.cif \
    ./data/cod/cif/2000000.cif
```

- Validate file `2000000.cif` against the `CIF_CORE`, `CIF_PD` and `CIF_COD` DDL1 dictionaries:

```
./cod-tools/bin/cif_validate \
    --ddl1-add-dictionary ./dictionaries/ddl1/IUCr/cif_core.dic \
    --ddl1-add-dictionary ./dictionaries/ddl1/IUCr/cif_pd.dic \
    --ddl1-add-dictionary ./dictionaries/ddl1/COD/cif_cod.dic
    ./data/cod/cif/2000000.cif
```

It should be noted that the order in which the DDL1 dictionaries are provided is important since it determines the dictionary merge order. The first DDL1 dictionary serves as the base while the remaining ones are merged sequentially using the OVERLAY mode as specified in the *International Tables for Crystallography Volume G* [1].

- Validate a CIF file read from the standard input stream against the `CIF_CORE` DDL1 dictionary:

```
cat ./data/cod/cif/1501972.cif | \
./cod-tools/bin/cif_validate \
    --ddl1-dictionaries ./dictionaries/ddl1/IUCr/cif_core.dic
```

- Validate all CIF files from the `./data/cod/cif/` directory against the `CIF_CORE`, `CIF_PD` and `CIF_COD` DDL1 dictionaries:

```
find ./data/cod/cif/ -name '*.cif' | \
xargs cif_validate \
    --ddl1-add-dictionary ./dictionaries/ddl1/IUCr/cif_core.dic \
    --ddl1-add-dictionary ./dictionaries/ddl1/IUCr/cif_pd.dic \
    --ddl1-add-dictionary ./dictionaries/ddl1/COD/cif_cod.dic
```

- Validate the `1501972.cif` file against the `CIF_CORE` and `CIF_COD` DDLm dictionaries. In this particular case the DDLm dictionary import path, that is required to correctly resolve internal dictionary import statements, is provided using the `--add-ddlm-import-path` command line option:

```
./cod-tools/bin/cif_validate \
    --ddlm-add-dictionary ./dictionaries/ddlm/IUCr/cif_core.dic \
    --ddlm-add-dictionary ./dictionaries/ddlm/COD/cif_cod.dic \
    --add-ddlm-import-path './dictionaries/ddlm/IUCr' \
    ./data/cod/cif/1501972.cif
```

- Validate the `1501972.cif` file against the `CIF_CORE` and `CIF_COD` DDLm dictionaries. In this particular case the DDLm dictionary import path, that is required to correctly resolve internal dictionary import statements, is provided using the `COD_TOOLS_DDLM_IMPORT_PATH` environment variable:

```
COD_TOOLS_DDLM_IMPORT_PATH=./dictionaries/ddlm/IUCr:${COD_TOOLS_DDLM_IMPORT_PATH}
export COD_TOOLS_DDLM_IMPORT_PATH

./cod-tools/bin/cif_validate \
    --ddlm-add-dictionary ./dictionaries/ddlm/IUCr/cif_core.dic \
    --ddlm-add-dictionary ./dictionaries/ddlm/COD/cif_cod.dic \
    ./data/cod/cif/1501972.cif
```

- Validate the `2000000.cif` file against the `CIF_CORE` and `CIF_CORE_RESTRAINTS` DDL1 dictionaries and the `CIF_COD` DDLm dictionary:

```
./cod-tools/bin/cif_validate \
    --ddl1-add-dictionary ./dictionaries/ddl1/IUCr/cif_core.dic \
    --ddl1-add-dictionary ./dictionaries/ddl1/IUCr/cif_core_restraints.dic \
    --ddlm-add-dictionary ./dictionaries/ddlm/COD/cif_cod.dic \
    --add-ddlm-import-path './dictionaries/ddlm/IUCr' \
    ./data/cod/cif/2000000.cif
```

  It should be noted, that the validator does not merge DDL1 and DDLm dictionaries into a single virtual dictionary and instead validates against DDL1 and DDLm dictionaries using two separate validation workflows.

- Validate the `1000000.cif` file against the `CIF_CORE` DDLm dictionary. In this particular case the `CIF_CORE` dictionary is provided using the generic `--add-dictionary` option that does not explicitly specify the dictionary DDL type:

```
./cod-tools/bin/cif_validate \
    --add-dictionary ./dictionaries/ddlm/IUCr/cif_core.dic \
    ./data/cod/cif/1000000.cif
```

  The use of the `--add-dictionary` option is discouraged and should only be used when the dictionary DDL type is not known in advance.

The `cif_validate` program outputs validation issues to the standard output stream (*stdout*) as single-line human-readable warning messages that follow the same formal syntax as the warning messages issued by the `COD::CIF::Parser` [2], i.e.:

```
> ./cod-tools/bin/cif_validate: ./data/cod/cif/1501972.cif data_1501972: NOTE, data item
  '_diffrn_source_current' value '40_mA' violates type constraints -- the value should be a numerically
  interpretable string, e.g. '42', '42.00', '4200E-2'.
> ./cod-tools/bin/cif_validate: ./data/cod/cif/1501972.cif data_1501972: NOTE, data item
  '_exptl_crystal_density_diffrn' value '1.66627(10)' is not permitted to contain the appended standard
  uncertainty value '(10)'.
```

Please note, that the program output in the provided example has been slightly modified for readability by marking the beginning of each new line with the greater-than (“>”) symbol.

```
.
├── cod-tools
│   └── ...
├── data
│   └── cod
│       └── cif
│           ├── 1000000.cif
│           ├── 1501972.cif
│           ├── 2000000.cif
│           ├── 2000002.cif
│           └── 4000001.cif
└── dictionaries
    ├── ddl1
    │   ├── COD
    │   │   └── cif_cod.dic
    │   └── IUCr
    │       ├── cif_core.dic
    │       ├── cif_core_restraints.dic
    │       └── cif_pd.dic
    └── ddlm
        ├── COD
        │   └── cif_cod.dic
        └── IUCr
            ├── cif_core.dic
            ├── templ_attr.cif
            └── templ_enum.cif
```

Figure S1: The directory layout of files that are used in the provided `cif_validate` program usage examples. The `cod-tools` directory contains the local installation of the `cod-tools` package. The contents of the `cod-tools` directory were replaced with an ellipsis ("...").

3 Usage examples of programs from the `cod-tools` software package

All programs described in this section provide an interface of a Unix filter. That is, the input CIF files are read from the provided locations in the file system or the standard input stream (*stdin*) and the modified CIF files are output to the standard output stream (*stdout*). Errors, warnings and notes produced while handling the input files are output to the standard error stream (*stderr*) and follow the same formal syntax as the warning messages produced by the `COD::CIF::Parser` [2].

Each program description is accompanied by one or more usage examples which consist of the following sections:

- **Input CIF file (`input.cif`)**. Contains the contents of the input CIF file that is used in the example.
- **Syntax errors**. Contains syntax error messages that are generated by the `COD::CIF::Parser` while processing the input CIF file.
- **Validation messages**. Contains validation messages that are generated by the `cif_validate` program while validating the input CIF file against the DDL1 `CIF_CORE` dictionary.
- **Explanation**. Contains a more detailed description of the syntax or semantic issues in the input CIF file.
- **Command**. Contains a `Bash` shell command that can be used under the Ubuntu 20.04 GNU/Linux system to correct the input CIF file.
- ***stderr***. Contains errors, warnings and notes that are output to *stderr* while running the command;
- ***stdout***. Contains the corrected input CIF file that is output to *stdout* while running the command.

Text in the **Syntax errors**, **Validation messages** and ***stderr*** sections is folded for readability. The start of each new line in these sections is marked with the “>” symbol.

3.1 `utf8-to-cif`

The `utf8-to-cif` program converts UTF-8 text to a form that is compatible with the CIF 1.1 data format. Unicode characters that fall outside of the CIF 1.1 character set are preferably expressed as CIF 1.1 special codes with hexadecimal numeric character references being used as a fallback mechanism. Since the use of numeric character references is not a universally accepted approach when dealing with CIF 1.1 files, CIF handling programs that were not developed by the COD team are unlikely to place any special meaning on these references. The program can be used as the initial step in the CIF processing pipeline to avoid syntax errors that may be caused by improperly expressed UTF-8 characters.

3.1.1 Usage example 1: conversion of Unicode characters to CIF 1.1 special codes

- **Input CIF file (`input.cif`):**

```
#\#CIF_1.1
data_charset_test
loop_
_publ_author_name
'Röntgen,␣Wilhelm␣Conrad'
```

- **Syntax errors:**

```
> ./cod-tools/bin/cif_validate: input.cif(5,1) data_charset_test: ERROR, incorrect CIF syntax:
> 'Röntgen,␣Wilhelm␣Conrad'
> ^
```

- **Explanation:**

  The character set of CIF 1.1 files is limited to a subset of the ASCII character set. In this particular case, the author name contains an accented character (“ö”) that needs to be replaced with a special code in order to properly record it in the CIF 1.1 file.

- **Command:**

```
./cod-tools/bin/utf8-to-cif input.cif
```

- ***stdout***:

```
#\#CIF_1.1
data_charset_test
loop_
_publ_author_name
'R\"ontgen,␣Wilhelm␣Conrad'
```

3.2 cif_fix_values

The `cif_fix_values` program resolves various simple semantic issues in CIF files. The program can regularise the values of various temperature data items (i.e. the `_chemical_melting_point`), correct values of the `_exptl_crystal_density_meas` data item, correct misspelt values by consulting a built-in table or an external replacement list file as well as carry out various other minor corrections. Upon successful termination, a summary of corrections that were applied to the input CIF file is output to the *stderr* as well as recorded in the output CIF file using the `_cod_depositor_comments` data item.

3.2.1 Usage example 1: correction of misspelt enumeration values

- **Input CIF file (`input.cif`):**

```
data_enumeration_test
_exptl_absorpt_correction_type 'MULTI␣SCAN'
```

- **Validation messages:**

```
> ./cod-tools/bin/cif_validate: test.cif data_enumeration_test:
  NOTE, data item '_exptl_absorpt_correction_type' value 'MULTI␣SCAN' must be one of
  the enumeration values ['analytical', 'cylinder', 'empirical', 'gaussian', 'integration',
  'multi-scan', 'none', 'numerical', 'psi-scan', 'refdelf', 'sphere'].
```

- **Explanation:**

  Enumeration values are case sensitive and must appear exactly as they are defined in the dictionary. In this particular case the enumeration value is written in upper case instead of lower case and does not contain a hyphen (i.e. "MULTI SCAN" instead of "multi-scan").

- **Command:**

```
./cod-tools/bin/cif_fix_values input.cif --fix-enums
```

- ***stderr***:

```
> ./cod-tools/bin/cif_fix_values: input.cif data_enumeration_test:
  NOTE, data item '_exptl_absorpt_correction_type' value 'MULTI␣SCAN' was changed to 'multi-scan'
  in accordance with the built-in table derived from the CIF Core dictionary named 'cif_core.dic'
  version 2.4.5 last updated on 2014-11-21.
```

- ***stdout***:

```
data_enumeration_test
_exptl_absorpt_correction_type multi-scan
_cod_depositor_comments
;
The following automatic conversions were performed:

data item '_exptl_absorpt_correction_type' value 'MULTI␣SCAN' was
changed to 'multi-scan' in accordance with the built-in table derived
from the CIF Core dictionary named 'cif_core.dic' version 2.4.5 last
updated on 2014-11-21.

Automatic conversion script
Id: cif_fix_values 8533 2020-09-29 07:54:47Z antanas
;
```

3.2.2 Usage example 2: correction of temperature values

- **Input CIF file (`input.cif`):**

```
data_melting_point_test
_chemical_melting_point '145␣C'
```

- **Validation messages:**

```
> ./cod-tools/bin/cif_validate: test.cif data_melting_point_test:
  NOTE, data item '_chemical_melting_point' value '145␣C' violates type constraints --
  the value should be a numerically interpretable string, e.g. '42', '42.00', '4200E-2'.
```

- **Explanation:**

  The definition of the `_chemical_melting_point` data item in the `CIF_CORE` dictionary states that the item should only have numeric values. The same definition also specifies that the recorded temperature should be expressed in kelvins. In this particular case the data item value contains a superfluous unit designator ("C") and is also incorrectly expressed in degrees Celsius instead of kelvins.

- **Command:**

```
./cod-tools/bin/cif_fix_values input.cif --fix-temperature
```

- ***stderr*:**

```
> ./cod-tools/bin/cif_fix_values: input.cif data_melting_point_test:
  NOTE, data item '_chemical_melting_point' value '145␣C' was changed to '418.15' --
  it was converted from degrees Celsius (C) to kelvins (K).
```

- ***stdout*:**

```
data_melting_point_test
_chemical_melting_point 418.15
_cod_depositor_comments
;
The following automatic conversions were performed:

data item '_chemical_melting_point' value '145␣C' was changed to
'418.15' -- it was converted from degrees Celsius (C) to kelvins (K).

Automatic conversion script
Id: cif_fix_values 8533 2020-09-29 07:54:47Z antanas
;
```

3.2.3 Usage example 3: correction of density values

- **Input CIF file (`input.cif`):**

```
data_crystal_density_test
_exptl_crystal_density_meas None
```

- **Validation messages:**

```
> ./cod-tools/bin/cif_validate: test.cif data_crystal_density_test:
  NOTE, data item '_exptl_crystal_density_meas' value 'None' violates type constraints --
  the value should be a numerically interpretable string, e.g. '42', '42.00', '4200E-2'.
```

- **Explanation:**

  The definition of the `_exptl_crystal_density_meas` data item in the `CIF_CORE` dictionary states that the item should only have numeric values. The same definition also specifies that the recorded density should be expressed in megagrams per cubic metre. In this particular case the data item value is a text string that implies that the density was not measured at all ("None").

- **Command:**

```
./cod-tools/bin/cif_fix_values input.cif --fix-density-meas
```

- ***stderr***:

```
> ./cod-tools/bin/cif_fix_values: input.cif data_crystal_density_test:
  NOTE, data item '_exptl_crystal_density_meas' value 'None' was changed to '?' --
  the value is perceived as not measured.
```

- ***stdout***:

```
data_crystal_density_test
_exptl_crystal_density_meas ?
_cod_depositor_comments
;
The following automatic conversions were performed:

data item '_exptl_crystal_density_meas' value 'None' was changed to
'?' -- the value is perceived as not measured.

Automatic conversion script
Id: cif_fix_values 8533 2020-09-29 07:54:47Z antanas
;
```

3.3 `cif_correct_tags`

The `cif_correct_tags` program corrects misspelt data names in CIF files. The program can restore the proper data name by applying several ad-hoc rules and by consulting a built-in table or an external replacement list file. Upon successful termination, a summary of corrections that were applied to the input CIF file is output to the *stderr* as well as recorded in the output CIF file using the `_cod_depositor_comments` data item.

The replacement list file maps common misspelt data name variants to the proper data names that they should be replaced by. The file consists of three types of lines:

- ***Replacement pair lines*** each of which contains a misspelt data name and a properly spelt data name that the former should be replaced by. Data names must be separated by one or more whitespace symbols. Trailing and leading whitespace symbols are silently ignored.

- ***Comment lines*** that start with the hash symbol ("#") and contain human-readable explanatory text. Comment lines are silently ignored.

- ***Whitespace lines*** that consist of 0 or more whitespace symbols. Whitespace lines are silently ignored.

An excerpt from the replacement list file used in the COD data maintenance operations is provided below:

```
#
# _incorrect_tag _correct_tag
#

# _geom_angle_atom_site_label_1
_geom_angle_atom_site_label_a _geom_angle_atom_site_label_1
_geom_angle_site_label_1 _geom_angle_atom_site_label_1
_geom_angle_atom_site_symbol_1 _geom_angle_atom_site_label_1

# _symmetry_Int_Tables
_symmetry_intl_tables_no _symmetry_Int_Tables_number
_symmetry_inl_tables_number _symmetry_Int_Tables_number
_symmetrry_inl_tables_number _symmetry_Int_Tables_number
_symmetry_intl_tables_number _symmetry_Int_Tables_number
```

The full replacement list file can be retrieved from the `cod-tools` Subversion repository (see section 1.1) by executing the following command:

```
svn export svn://www.crystallography.net/cod-tools/tags/v3.0.0/data/replacement-values/replacement_tags.lst
```

3.3.1 Usage example 1: correction of misspelt data items using a replacement list file

- **Input CIF file (`input.cif`):**

```
data_misspelt_data_name_test
__space_group_crystal_system orthorhombic
_space_group_int_number 42
_space_group_h-m 'F␣m␣m␣2'
```

- **Replacement list file (`replacement.lst`):**

```
# Comment lines start with the '#' symbol
_space_group_int_number _space_group_IT_number
_space_group_h-m _space_group_name_H-M_alt
```

- **Validation messages:**

```
> ./cod-tools/bin/cif_validate: test.cif data_misspelt_data_name_test:
  NOTE, definition of the '__space_group_crystal_system' data item was not found in
  the provided dictionaries.
> ./cod-tools/bin/cif_validate: test.cif data_misspelt_data_name_test:
  NOTE, definition of the '_space_group_h-m' data item was not found in
  the provided dictionaries.
> ./cod-tools/bin/cif_validate: test.cif data_misspelt_data_name_test:
  NOTE, definition of the '_space_group_int_number' data item was not found in
  the provided dictionaries.
```

- **Explanation:**

  It is highly recommended that all data items used in a CIF file are properly formally defined in DDL dictionaries. While data items from dictionaries that are private, difficult to obtain or non-existent at all (i.e. `_shelx_*`, `_olex2_*`, `_jana_*`) are routinely encountered in modern CIF files, it is nevertheless recommended to inspect all unrecognised data names for spelling mistakes. In this particular case one of the reported data names (`__space_group_crystal_system`) contains a simple spelling mistake that can be corrected by applying ad-hoc rules and consulting a built-in table while the remaining two contain more serious spelling mistakes, correction of which require the use of the `replacement.lst` replacement list file.

- **Command:**

```
./cod-tools/bin/cif_correct_tags input.cif --replacement-list replacement.lst
```

- ***stderr*:**

```
> ./cod-tools/bin/cif_correct_tags: input.cif data_misspelt_data_name_test:
  NOTE, data name '__space_group_crystal_system' was replaced with '_space_group_crystal_system'.
> ./cod-tools/bin/cif_correct_tags: input.cif data_misspelt_data_name_test:
  NOTE, data name '_space_group_int_number' was replaced with '_space_group_IT_number'
  as specified in the replacement file 'replacement.lst'.
> ./cod-tools/bin/cif_correct_tags: input.cif data_misspelt_data_name_test:
  NOTE, data name '_space_group_h-m' was replaced with '_space_group_name_H-M_alt'
  as specified in the replacement file 'replacement.lst'.
```

- ***stdout***:

```
data_misspelt_data_name_test
_space_group_crystal_system orthorhombic
_space_group_IT_number 42
_space_group_name_H-M_alt 'F␣m␣m␣2'
_cod_depositor_comments
;
The following automatic conversions were performed:

data name '__space_group_crystal_system' was replaced with
'_space_group_crystal_system'.

data name '_space_group_int_number' was replaced with
'_space_group_IT_number' as specified in the replacement file
'replacement.lst'.

data name '_space_group_h-m' was replaced with
'_space_group_name_H-M_alt' as specified in the replacement file
'replacement.lst'.

Automatic conversion script
Id: cif_correct_tags 8533 2020-09-29 07:54:47Z antanas
;
```

4 The `cod_validation` database

COD entry validation issues are stored on the `sql.crystallography.net` server using `MariaDB` DBMS that is compatible with most modern `MySQL` clients. The database can be accessed using the passwordless `cod_reader` user that has `SELECT` privileges on all tables in the database. For example, running the following command under Ubuntu 20.04 GNU/Linux system should return a listing of all of the available database tables:

```
mysql -u cod_reader -h sql.crystallography.net cod_validation -e 'SHOW␣TABLES;'
```

Version `1.0.0` of the `cod_validation` database schema contains 3 tables (see Fig. S2). The `version` table identifies the currently used database schema, `entry_validation_state` table mostly contains information used to identify COD entries that need revalidation while the `validation_issue` table contains the validation issue descriptions. For most applications the `validation_issue` table should be sufficient.

4.1 `RestfulDB` based web interface

The `RestfulDB` software provides a web GUI as well as a RESTful API for interaction with the `cod_validation` SQL database. Notable `RestfulDB` endpoints:

- `https://sql.crystallography.net/db/cod_validation` – the `cod_validation` database endpoint that provides the list of all available database tables;
- `https://sql.crystallography.net/db/cod_validation/validation_issue` – the `validation_issue` table endpoint that provides access to the table data in various formats such as HTML, CSV, JSON, ODT, etc. The table endpoint also provides additional data processing functionality such as data sorting and filtering.

4.2 SQL query examples

SQL queries provided in this section are executed while logged into the `cod_validation` database as the `cod_reader` user. This can be achieved by running the following command:

```
mysql -u cod_reader -h sql.crystallography.net cod_validation
```

Examples of several useful SQL queries:

1. Select the number of COD entries that have associated validation issues:

```
SELECT COUNT(DISTINCT `cod_id`)
FROM `validation_issue`;
```

2. Select the number of COD entries that have associated DDL1 validation issues:

```
SELECT COUNT(DISTINCT `cod_id`)
FROM `validation_issue`
WHERE `validation_type` = 'DDL1';
```

3. Select the number of COD entries that have associated DDLm validation issues:

```
SELECT COUNT(DISTINCT `cod_id`)
FROM `validation_issue`
WHERE `validation_type` = 'DDLm';
```

4. Select all DDL1 validation messages associated with COD entry 1000000:

```
SELECT `message`
FROM `validation_issue`
WHERE `cod_id` = '1000000'
AND `validation_type` = 'DDL1';
```

5. Select 10 most common DDLm validation messages:

```
SELECT `message`, COUNT(*)
FROM `validation_issue`
WHERE `validation_type` = 'DDLm'
GROUP BY `message`
ORDER BY COUNT(*) DESC
LIMIT 10;
```

6. Select 5 most common validation messages that report incorrect `_exptl_absorpt_correction_type` data item values:

```
SELECT `message`, COUNT(*)
FROM `validation_issue`
WHERE `message` LIKE
    "%␣'_exptl_absorpt_correction_type'␣value␣'%'␣must␣be%"
GROUP BY `message`
ORDER BY COUNT(*)
DESC LIMIT 5;
```

7. Select the number of COD entries that have associated DDLm validation issues that do not involve top level list containers or missing category keys:

```
SELECT COUNT(DISTINCT `cod_id`)
FROM `validation_issue`
WHERE `validation_type` = 'DDLm'
AND `message` NOT LIKE
    "%top␣level␣list␣container%"
AND `message` NOT LIKE
    "missing␣category␣key%";
```

8. Select the number of COD entries that have associated DDL1 validation issues that do not involve missing looped list keys or missing mandatory data items:

```
SELECT COUNT(DISTINCT `cod_id`)
FROM `validation_issue`
WHERE `validation_type` = 'DDL1'
AND `message` NOT LIKE
    "missing␣looped␣list␣reference%"
AND `message` NOT LIKE
    "%mandatory%";
```

```
-- -----------------------------------------------------
-- Schema cod_validation
-- -----------------------------------------------------


-- -----------------------------------------------------
-- Table `version`
-- -----------------------------------------------------
CREATE TABLE IF NOT EXISTS `version` (
  `id` INT(11) UNSIGNED NOT NULL AUTO_INCREMENT
     COMMENT 'Artificial␣primary␣key␣of␣the␣table',
  `number` CHAR(10) CHARACTER SET 'ascii' NOT NULL
     COMMENT 'Version␣of␣the␣database␣schema',
  `schema_url` VARCHAR(255) NULL DEFAULT NULL
     COMMENT 'URL␣of␣the␣database␣schema',
  PRIMARY KEY (`id`),
  UNIQUE INDEX `timestamp_UNIQUE` (`timestamp` ASC),
  UNIQUE INDEX `number_UNIQUE` (`number` ASC))
DEFAULT CHARACTER SET = utf8mb4
COMMENT = 'Database␣schema␣version';


-- -----------------------------------------------------
-- Table `entry_validation_state`
-- -----------------------------------------------------
CREATE TABLE IF NOT EXISTS `entry_validation_state` (
  `id` INT UNSIGNED NOT NULL AUTO_INCREMENT
     COMMENT 'Artificial␣primary␣key␣of␣the␣table',
  `cod_id` MEDIUMINT(7) UNSIGNED NOT NULL
     COMMENT 'COD␣ID␣of␣the␣validated␣entry',
  `entry_svn_revision` INT UNSIGNED NOT NULL
     COMMENT 'SVN␣revision␣of␣the␣validated␣COD␣entry',
  `validation_setup_version` VARCHAR(32) CHARACTER SET 'ascii' NOT NULL
     COMMENT 'Version␣number␣of␣the␣entire␣validation␣setup',
  `validation_timestamp` TIMESTAMP NOT NULL DEFAULT CURRENT_TIMESTAMP
     COMMENT 'Timestamp␣of␣the␣most␣recent␣entry␣validation',
  `is_revalidation_required` TINYINT(1) UNSIGNED NOT NULL
     COMMENT 'Boolean␣value␣denoting␣if␣the␣entry␣should␣be␣revalidated',
  UNIQUE INDEX (`cod_id` ASC),
  PRIMARY KEY (`id`))
DEFAULT CHARACTER SET = utf8mb4
COMMENT = 'Validation␣state␣of␣each␣COD␣entry';


-- -----------------------------------------------------
-- Table `validation_issue`
-- -----------------------------------------------------
CREATE TABLE IF NOT EXISTS `validation_issue` (
  `id` INT UNSIGNED NOT NULL AUTO_INCREMENT
     COMMENT 'Artificial␣primary␣key␣of␣the␣table',
  `cod_id` MEDIUMINT(7) UNSIGNED NOT NULL
     COMMENT 'COD␣ID␣of␣the␣validated␣entry',
  `program_name` VARCHAR(255) NOT NULL
     COMMENT 'Name␣of␣the␣program␣that␣generated␣the␣message',
  `validation_type` ENUM('DDL1', 'DDLm')
     CHARACTER SET 'ascii' NOT NULL
     COMMENT 'General␣validation␣type␣of␣the␣issue',
  `issue_severity` ENUM('NOTE', 'WARNING', 'ERROR')
     CHARACTER SET 'ascii' NULL DEFAULT NULL
     COMMENT 'Severity␣level␣of␣the␣issue',
  `message` VARCHAR(1024) NOT NULL
     COMMENT 'Message␣describing␣the␣issue',
  PRIMARY KEY (`id`))
DEFAULT CHARACTER SET = utf8mb4
COMMENT = 'Issues␣generated␣by␣the␣COD␣CIF␣validation␣software';
```

Figure S2: The complete `cod_validation` database schema version `1.0.0`.

5 Distribution of distinct validation issues per each COD entry

The distributions of distinct DDL1 and DDLm validation issues per each COD entry from COD revision 249495 are provided in Fig. S3. In this analysis a distinct validation issue is defined as a validation issue that has a unique combination of validation issue type and the affected data items. For example, an entry that has 12 non-numeric `_atom_site.occupancy` data item values would be considered as having 1 distinct validation issue while an entry with 2 non-numeric `_atom_site.occupancy` data item values, 4 out-of-range `_atom_site.occupancy` data item values and 5 non-numeric `_atom_site.fract_x` data item values would be considered as having 3 distinct validation issues.

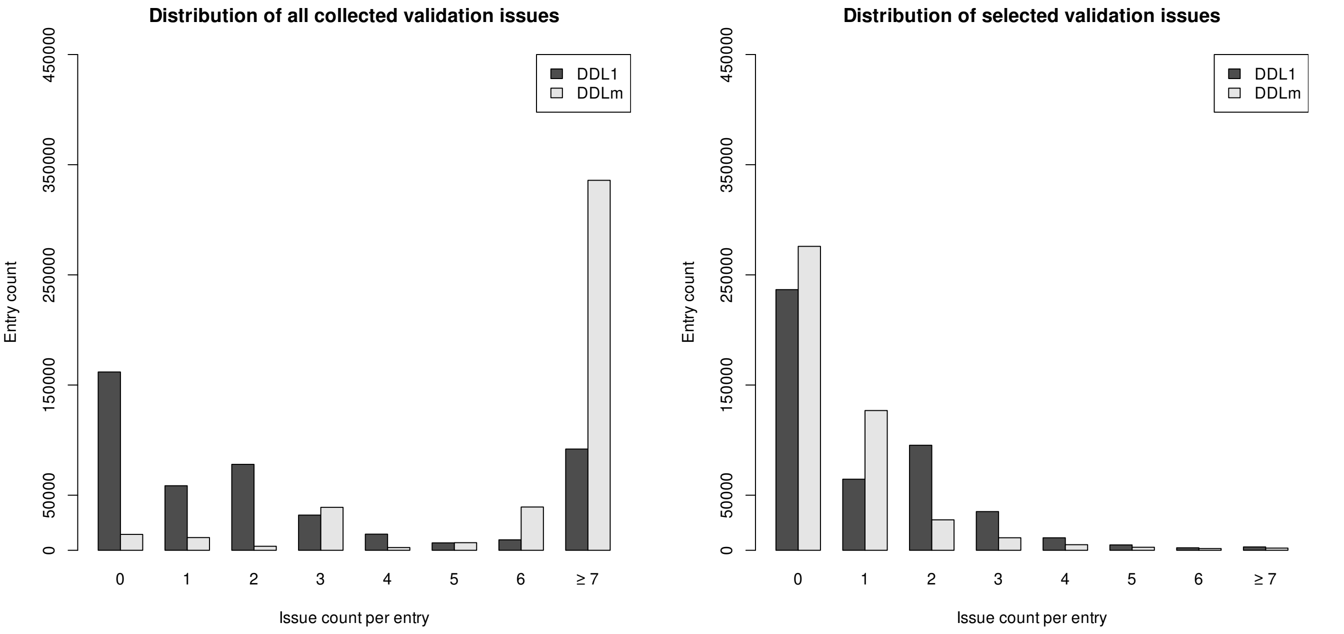

Figure S3: The distributions of distinct validation issues per each entry from COD revision 249495. The bar chart on the left was generated from all collected validation issues while the bar chart on the right was generated after removing validation issues involving unrecognised data item names, missing category keys and complex data structures. The excluded validation issues are extremely useful in detecting more obscure data anomalies, however, they do not generally prevent the affected files from being used for most applications.

References

[1] S. R. Hall and B. McMahon, eds., *International Tables for Crystallography*, vol. G. International Union of Crystallography, 2006.

[2] A. Merkys, A. Vaitkus, J. Butkus, M. Okulič-Kazarinas, V. Kairys, and S. Gražulis, "*COD::CIF::Parser*: an error-correcting CIF parser for the Perl language", *Journal of Applied Crystallography*, vol. 49, no. 1, pp. 292–301, 2016.
